# Metabolic syndrome and prostatic disease: potentially role of polyphenols in preventive strategies. A review

**DOI:** 10.1590/S1677-5538.IBJU.2015.0095

**Published:** 2016

**Authors:** Tommaso Castelli, Giorgio Ivan Russo, Giulio Reale, Salvatore Privitera, Mario Chisari, Eugenia Fragalà, Vincenzo Favilla, Sebastiano Cimino, Giuseppe Morgia

**Affiliations:** 1 Dipartimento di Urologia, Facoltà di Medicina Policlinico, Università di Catania, Italia

**Keywords:** Oxidative Stress, Prostatic Hyperplasia, Prostatic Neoplasms, Polyphenols

## Abstract

Benign prostatic hyperplasia and prostate cancer are two common urological diseases of the elderly. Scientific community has always looked for a link that could explain the correlation between the two diseases and the role of chronic inflammation in the pathogenesis of BPH and PCa. As shown by the reports of the two diseases relationship with oxidative stress and metabolic syndrome, the use of compounds with antioxidant action could therefore affect both the symptoms and their onset. Polyphenols appear to act not only against oxidative stress but also at different levels. The aim of this review is to evaluate the role of the most important polyphenols on these two urological diseases. As antioxidants these compounds seems to have a direct action on the cell cycle and hormone function, important for both prostate cancer and BPH. Despite a large number of articles about the relationship of the polyphenols with prostate cancer, very little evidence exists for BPH. Additional clinical trials or meta-analysis are necessary on this topic.

## INTRODUCTION

Benign prostatic hyperplasia (BPH) is one of the most frequent causes of Lower Urinary Tract Symptoms (LUTS) in men and about 50% of men between 50 and 60 years suffer from this disease (1). Even today, the exact molecular mechanisms underlying the development and progression of LUTS/BPH have not been fully understood. Certainly, recent studies have shown that chronic inflammation represents a crucial component in the pathogenesis of BPH, probably determining hyperplasia of prostate cells. Inflammatory cells in fact, produce growth factors such as vascular endothelial growth factors (VEGF) or tumor growth factor-β (TGF-β), which can support the fibromuscular growth in BPH (2-4). The etiology of BPH is still far from being fully understood but multiple partially overlapping and complementary theories have been proposed (5). Very recently, epidemiologic and clinical studies have provided emerging evidences of a possible role of metabolic syndrome (MetS) and its components in benign prostatic hyperplasia (BPH) and related lower urinary tract symptoms (LUTS) (6, 7). MetS can broadly be considered a systemic inflammatory state and a chronic inflammation driven tissue remodeling, including BPH pathogenesis (8). A recent review summarized a direct and significant relationship between some components of MetS (obesity, high dyslipidemia, insulin resistance, and hypertension) and the BPH-LUTS complex. Furthermore, recent evidences suggested that severity of BPH-LUTS is strictly associated with increase in the number of components of MetS (9). The influence of dietary fat on BPH has been linked to specific fatty acids (FAs). In vivo studies have indicated that low-fat diets high in omega-3 polyunsaturated FAs reduce the development of prostatic disease. The omega-3 polyunsaturated FA serum composition was significantly decreased in patients with BPH (10). MetS is often characterized by oxidative stress that is involved in the pathogenesis of a variety of human diseases including atherosclerosis, diabetes, hypertension, aging, and cancer. The level of oxidative stress increases during aging and it could be related to prostatic diseases (11, 12). There are some evidences that prostatic inflammation could be a key component in BPH and BPH progression.

The level of oxidative stress increases during aging and could be either because of an increased production of reactive oxygen species or a reduced ability to scavenge them. High glucose concentrations increase oxidative stress, in part by down regulating catalase and mitochondrial superoxide expression, leading to a higher risk of insulin resistance (13). Recent studies suggest that oxidative stress and hyperinsulinemia, secondary to insulin resistance, are risk factors for cell proliferation and cell remodeling present in BPH. Insulin resistance can also lead to dyslipidemia characterized by high triglyceride and low high-density lipoprotein cholesterol (HDL-C) levels. Abdominal obesity is commonly associated with the development of vascular diseases, insulin resistance, and associated complications. Excessive visceral and subcutaneous fat increase oxidative stress and simultaneously decrease the expression and activity of key cytoprotective enzymes, including the heme oxygenase (HO) system (14). We have evaluated the activity of Heme oxygenases system in patients affected by BPH and found that low HDL-C and high triglyceride levels significantly affected HO-1 and HO-2 prostatic levels, with consequent increase of oxidative stress and remodeling of prostate tissue (7). Obesity-mediated adipocyte dysfunction may impact the function of other organs, including the prostate.

A major clinical study on BPH (Reduce study) recently demonstrated the link between histological prostatic inflammation and prostate enlargement or symptoms scores (15). Numerous major key players in chronic inflammation have been studied in BPH: varieties of growth factors and cytokines have been shown to be involved both in the inflammatory process and in the epithelial/stromal prostatic cells interactions (16). These mediators are released in the prostatic gland by inflammatory cells that can be found on most of the surgery-derived BPH specimens (17). The inflammatory cells may trigger a sophisticated and well-orchestrated inflammatory cascade, resulting in excessive oxidative stress, activation of the transcription factor nuclear factor-kappa B (NF-κB), production of several cytokines and overexpression of inducible-cyclooxygenase (COX-2), inducible-nitric-oxide-synthase (iNOS) and 5-lipoxygenase (5-LOX), leading, in turn, to the release of prostaglandins, nitrates, and leukotrienes. Furthermore, inflammatory cells produce growth factors, such as vascular endothelial growth factor (VEGF) and transforming growth factor-β (TGF-β), which may support fibromuscular growth in BPH (18).

Also for prostate cancer is described in literature the possibility that the pathogenesis of the disorder is linked to chronic inflammation: as disclosed by De Nunzio et al. the presence of oxidative stress associated to chronic inflammation in the cellular environment causes an increase of pro-inflammatory cytokines and growth factors, which determine an increase of the speed of cell replication, and therefore the possibility of incurring mutations (19). Furthermore according to the authors, the presence of PIA (proliferative inflammatory atrophy) precursor HGPIN and prostate cancer charged to the prostatic parenchyma, involves the presence of abnormal values of GSTP1 (gene coding for glutathione S-transferase), GSTA1 (gene coding for glutathione S-transferase A1) and COX-2 (enzyme determining the conversion of arachidonic acid (AA) in the prostaglandin endoperoxide H2 precursor of PGD2, PGE2, PGF2α, PGI2 and thromboxane A2).

Therefore, if a correlation between prostate diseases and oxidative stress or chronic inflammation could hypothesized, the use of components with exhibit antioxidant action could determine not only an improvement of prostatic symptoms but also counteract pathogenesis.

The aim of this review is to understand the role of polyphenols in prostatic diseases and their preventive role in preventive strategies.

## MATERIALS AND METHODS

This analysis was conducted according to the Preferred Reporting Items for Systematic Reviews and Meta-analysis guidelines (20). An electronic search of the Medline and Embase was undertaken until September 2014. The search was limited to English-Language articles. The search terms included prostate, benign prostatic hyperplasia, benign prostatic enlargement, metabolic syndrome, prostate volume, insulin resistance, obesity, hypertension, triglycerides, cholesterol, lower urinary tract symptoms, polyphenols, oxidative stress, anti-oxidant, prevention. Citation lists of retrieved articles were screened manually to ensure sensitivity of the search strategy. References of the included papers were hand searched to identify other potential relevant studies.

**Figure 1:**
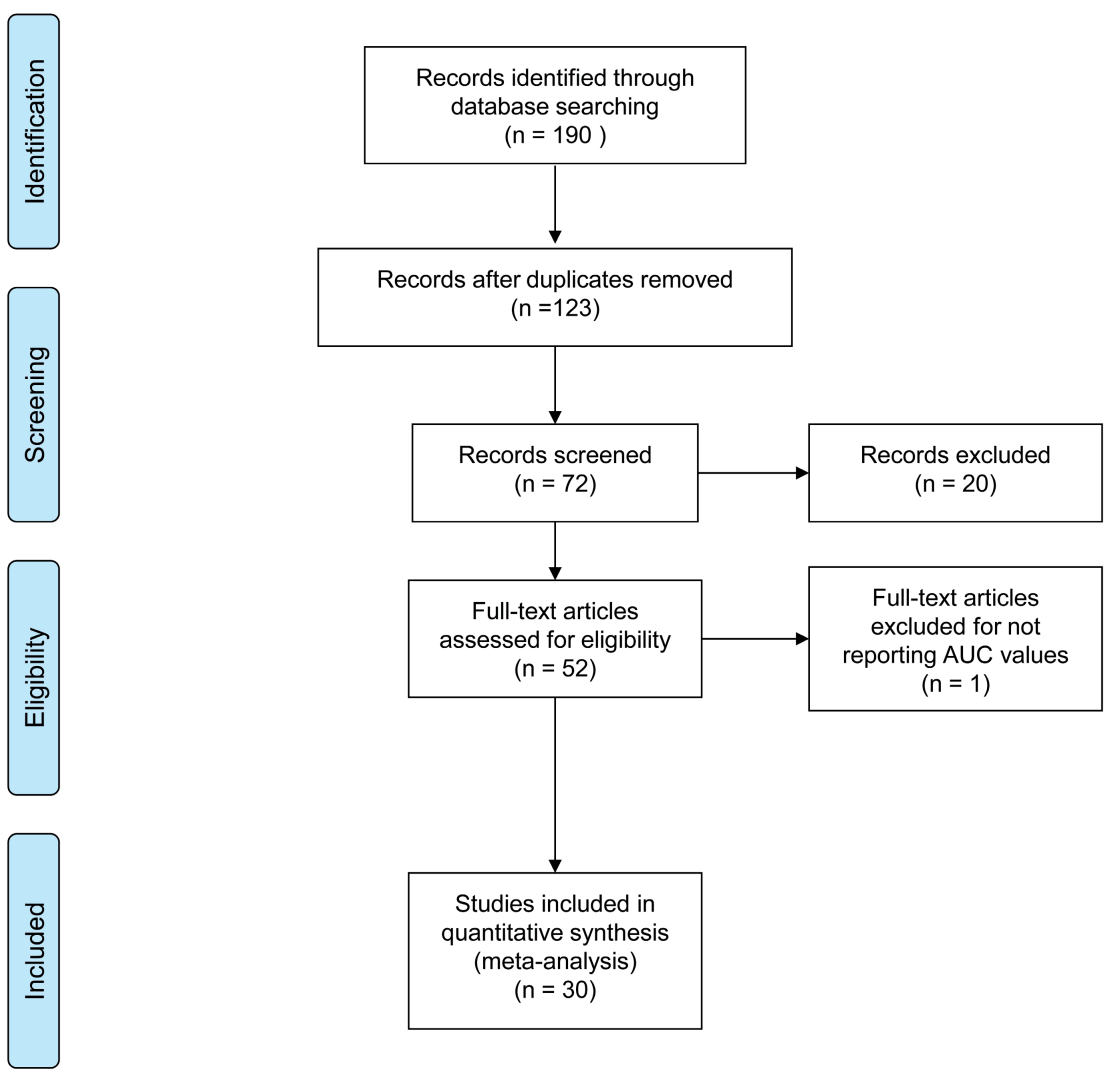
Flow Diagram of included studies.

Studies were reviewed by two independent reviewers (G.I.R. and G.R.); differences in opinion were discussed in consultation with the last author (G.M.). Figure-1 shows the flowchart of included studies. Table-1 lists the characteristics of included studies.

**Table 1:** Characteristics of selected study.

Polyphenols and prostate cancer					

Compound	Type of study	Cell culture system or animal studies	Concentration used		Mechanisms of action
Epigallocatechin-3-gallate (23, 24, 28,30,31,32,34,36)	In humans (23, 24, 29,30,36) In vivo (32,34) In vitro (28,33)	LNCaP (28,33) PC-3, and CWR22Rnu1 (28) LAPC-4 (33) TRAMP (32,34)	EGCG (10-40 micromol/L) (28) 0.06% EGCG in tap water (32) Solution of 0.1% green tea polyphenols EGCG (62%) (34) Green tea extract capsules ,250 mg twice daily (36)		Action on transcription factor Nf-kB inducing oxidative stress and downregolation of p53 (23, 24, 28, 29, 30) -antagonize the activity of IGF-1 and induce an receptorial antagonism for IGF-1 receptor (32,34) -inactivator of COX-2 (24, 31)
Curcumin	In vitro (40,42,43)	LNCaP (40,42) PC-3 (42,43)	2-4 mg/L (about 5-10μM) (40) 15 µM (43)		Increased the ratio of Bax to Bcl-2 proteins, decreased the activation of NFκB, PI3K/Akt and Stat3 pathways and cell migration (40) Down-regulation of transactivation and expression of AR, AP-1, NF-kB, and CREB-binding protein (CBP) (42) Inhibition of the IκB-kinase ,reduction in expression of CXCL1 and -2 ,downregulation of several important metastasis-promoting factors like COX2, SPARC and EFEMP (43)
Resveratrol	In vitro (45,46)	LNCaP (45,46,47) PC3 (45,46 ) and DU145 (45,46,47)	2–40 μM (46); 1 µmol/L (47)		Production of NO (45) Formation of free radicals (46) p53 activation and apoptosis (47)

Polyphenols and BPH					

Compound	Type of study	N° patients	Concentration used	Outcome	

Isoflavones (53)	In humans (53,55)	176 patients with	40 mg of isoflavones (53)	Superiority of isoflavones over placebo over 12 months[53]	
Isoflavones and lignans (55)		BPH (53) 25 patients (55)		Isoflavones, but not lignans, have some influence the benign prostatic growth (55)	

## POLYPHENOLS AND PROSTATE CANCER

From a careful analysis of the literature there are several studies that evaluated the use of polyphenols in the treatment of prostate cancer both in terms of primary prevention and secondary prevention of cancer. Polyphenols occur naturally in different foods, and today there are more than 8000 divided into different subclasses, of which the most represented are flavonoids stilbenes phenolic acids, and lignans (21). The importance of the polyphenols as anti-cancer substances shall be represented by fact that they possess properties that act at different levels (molecular and cellular), as described in several papers (21-24).

Among these compounds, as previously described, one of the most investigated is undoubtedly the green tea, the second most widely consumed beverage in the World after water (25). The tea plant (Camellia sinensis) has been cultivated in Asia for thousands of years and used for centuries in China, Japan, and Thailand as a traditional medicine with a variety of applications (26). Many beverages are rich of-epigallocatechin (EGC),-epicatechin-3-gallate (ECG) and-epicatechin (EC), gallic acid, chlorogenic acid, caffeic acid, like flavonols kaempferol, myricetin and quercetin and the most represented-epigallocatechin-3-gallate (EGCG), and several studies have investigated their role in the treatment of prostate cancer (24). To the best of our knowledge, particularly in prostate cancer cells, EGCG activates growth arrest and apoptosis primarily via p53-dependent pathway that involves the function of both p21 and Bax such that downregulation of either molecule confers a growth advantage to the cells. In androgen-sensitive LNCaP and androgen-insensitive PC-3 human prostate carcinoma cells, EGCG inhibited COX-2, (inducible enzymatic isoform, rapidly induced by growth factors, tumor promoters, oncogenes, and carcinogens) without affecting COX-1 expression at both the mRNA and protein levels (25, 27). Among the mechanism that make the polyphenols in green tea of primary importance in the treatment of prostate cancer is the action of the epigallocatechin-3-gallate as a transcription factor Nf-kB that underlies both inflammatory cellular processes that induce oxidative stress both anti-apoptotic events (downregulation p53) resulting in a cascade of events that leads to carcinogenesis: the activity of this factor is extremely elevated in tumor cells in patients with prostate cancer and this has been highlighted (23, 24, 28-30) as the epigallocatechin-3-gallate decreases the intracellular concentration of the protein by blocking the activity of the via connected to it.

The relationship between prostate cancer and the intake of green tea and then directly the action of polyphenols (in particular ‘epigallocatechin-3-gallate or EGCG) is expressed at different levels in prostate cancer etiology by modulating different pathways linked to inflammation chronic (action on NF-kB and COX-2) and also the metabolic syndrome itself (IGFR). Overexpression of NF-kB leads to the activation of several signaling pathways and among these also the activation of COX-2 which increases levels of pro-inflammatory cytokines. This determines as final result the pro oxidative imbalance and the formation of a eligible pabulum for carcinogenesis: to this regard it has been observed that the ECGC is also an inactivating of COX-2 without interfering with the activity of COX-1 (24, 31) supported by studies on transgenic mice (32). The anti-inflammatory action of ‘EGCG is also evident by its action on COX-2.

As reported by De Nunzio et al. (6) insulin resistance associated to metabolic syndrome, widely spread throughout the World, is manifested by an increased production of somatomedin C or IGF-1 receptor. Since the insulin like growth factor 1 has a potent mitogenic and anti-apoptotic action is evident how it is an important parameter for the development of cancer cells, and this process appears to be comparable to prostate cancer onset mechanism (33). It has been shown, in studies involving TRAMP mice (34) that EGCG is able to directly antagonize the activity of IGF-1 and induce a receptor antagonism for IGF-1 receptor (32).

Furthermore, other qualities have been recognized in the green tea and its phenolic component regarding prostate cancer: it was noted that the action of ECGC has not target only the proinflammatory pathways but also can play a role as antiandrogen. A review by Lecumberri et al. showed that ECGC has not only a direct role reducing testosterone levels, but also indirectly by inducing a receptor downregulation on prostate tissue in animal models (35). However, we must emphasize that studies in prostate cancer castration resistant patients showed no clinically valid results for the use of green tea (36).

Curcumin (diferuloylmethane) is a major chemical component of turmeric (Curcuma longa Linn.) and is used as a spice to give a specific flavor and yellow color to food in the Indian subcontinent (37). It has been used for centuries throughout Asia not only as a food additive but also as cosmetic and as a traditional herbal medicine to treat a variety of inflammatory conditions and chronic diseases. Over the past decade, several studies have substantiated the potential prophylactic or therapeutic value of curcumin and have unequivocally supported reports of its anti-carcinogenic properties, such as its ability to influence a diverse range of molecular targets within cells. To date, no studies have reported any toxicity associated with the use of curcumin in either animals or humans (38).

Likewise to ‘ECGC, many others polyphenols like curcumin exhibited anticancer/chemopreventive activity that is expressed at different levels. Wang et al. shown that the growth of human prostate cancer cell cultures is inhibited both by single administration curcumin (22%) or EGCG and (11%) or arctigenin (29%). However, they found that polyphenolic combination EGCG+curcumin, curcumin+arctigenin and curcumin+EGCG+arctigenin reduce tumor growth of cell lines respectively by 34%, 49% and 62% compared to control. The mechanism underlying these modifications, according to investigators, would be related primarily to an arrest of cell division of cancer cells in G1/G0 phase (directly related to the action of curcumin), to an increase of apoptotic factors Bcl-2/Bax (39) and to an inhibition via phosphorylation of nF-Kb (40). Other studies based on nanotechnology that administered curcumin to prostate cancer cell lines have shown that this method is more efficient than free curcumin to reduce the growth of cell colonies by arresting fosforilation of STAT 3 and dell’AKT, promoting the action of anti-apoptotic protein (Mcl-1, Bcl-xL) and inducing a PARP lysis. Additionally curcumin has antioxidant and anti-inflammatory mechanisms (41) and, likewise ECGC, has an activity which is expressed at the level of receptors androgens inducing downregulation of their own receptors (39, 42). The anti-tumor action of curcumin seems to be explained, according to Killian et al. (43,) also by the reduction process of metastatization of the cancer: in murin model it has been shown that in cell lines treated with curcumin there is a reduction of the genetic factors that promote the formation of lung metastases (SPARC (osteonectin), COX2 (PTGS2, prostaglandin-endoperoxidesynthase 2), ALDH3A1 (aldehyde dehydrogenase-3 family member A1) and EFEMP (EGF-containing fibulin-like extracellular matrix) through a reduction of the overexpression of the proteins CXCL1 and-2).

Resveratrol (trans-3, 4, 5-trihydroxystilbene, C14H12O3) is a plant-derived polyphenolic phytoalexin produced by the enzyme stilbene synthase in response to infection by the pathogen Botrytis cinerea and to a variety of stress conditions, such as vicissitudes in climate, exposure to ozone, sunlight and heavy metals. It exists in two isoforms: trans-resveratrol and cis-resveratrol where the trans-isomer is the more stable form. Resveratrol is present in red grapes, peanuts, some common drinks, and dietary supplements (44). Kampa et al. tested the anticancer ability of a mixture of polyphenols (catechin, epicatechin, quercetin, and resveratrol) extracted from wine on three prostate cancer cell lines: LNCaP and PC3 (hormone-sensitive), and DU145 (not hormone-sensitive) (45). They found that on LNCaP all three flavonoids were active (catechin, epicatechin, quercetin) and resveratrol was ineffective. On PC3 the action of the three flavonoids was more prominent and resveratrol acted only in high concentrations compared with DU145 which was more sensitive to resveratrol and not to the three flavonoids. As already noted for the other polyphenols, the mechanisms that explain the action of resveratrol are to be found in the different levels on which it performs the action of the various components of the wine: mechanisms of hormonal receptor antagonism concerning LNCaP and PC3 and molecular mechanism concerning DU145. Authors suggest that an important role of these molecules in question is represented by the balance of the antioxidant and NO production. Similar results were found in other in vitro studies on the same cell lines (LNCaP and, PC3 and concerning DU145) and has been shown that resveratrol, in addition to the mechanisms described above, would have inhibitory effect on the formation of free radicals in human macrophages, reducing oxidative stress within premalignant cells (46) and on the MAP kinase pathway (47).

## POLYPHENOLS AND BPH/LUTS

Despite numerous evidences that correlate benign prostatic hyperplasia, metabolic syndrome and oxidative stress (7, 8, 48-51), the correlation between polyphenols (high antioxidant dietary components) and BPH are still very limited. Little evidence can be found by searching each polyphenolic compound in relation to benign prostatic hyperplasia.

There are very few evidences in literature regarding green tea and its polyphenols (especially the ECGC): in a review Ranjan et al. (52) stated that the anti-proliferative activity of green tea antioxidant activity combined with the ‘action on 5a-reductase inhibitors may have a protective role both on the onset of BPH and on disease progression.

Certainly various studied correlated isoflavones and BPH: in a double-blinded, randomized controlled trial Wong et al. (53) examined 176 patients with BPH divided into two groups, one of which that was administered with isoflavones 40mg daily (n=88) and a placebo Group (n=88). Patient’s then underwent a control visit every three months during a 12 months period and were evaluated in terms of IPSS, Qmax, and 36-Item Short Form Health Survey (SF-36). At the end of 12 months, IPSS (SF-36) was not statistically significant between the two groups. The increase in Qmax (from 6 to 12 months) and incomplete emptying subscore in IPSS from baseline to the 12th month was slightly significant. The effect of this kind of polyphenols on BPH may be explained by its action on the prostatic parenchyma both on the epithelial tissue cell (throughout a 5α-reductase action and a uridine 5-diphospho-glucuronsyltransferase activation) and on the stromal cell (throughout aromatase inhibition, and estrogen receptor antagonism) (54). The authors emphasized that actually there are no indication for the use of isoflavons as therapy for BPH (54). Parenchymal level of isoflavons in BPH patients are lower than control, while blood level of isoflavons didn’t show any statistically difference (55).

## CONCLUSIONS

In conclusion, this review aimed to research which could be the use of polyphenols on BPH and prostate cancer. As revealed from the papers examined, the action of polyphenols is directed to different levels in the prostate cell: the primary action of these molecules is opposing to oxidative stress, but other evidences confirm that these compounds influence the growth of the prostate androgenic mediated cells and the cell cycle phases. Although a large amount of studies in vitro and in murin model have been conducted until now, few clinical trials, using precise concentrations of these compounds, have been performed. If we consider the relationship between polyphenols and BPH, we observe that there are very few studies carried out on this topic. Therefore, since the use of the polyphenols seem to have good perspectives, randomized clinical trials and meta-analyzes are needed in patients with prostate cancer and especially in patients with BPH.
